# Synthesis and homopolymerization kinetics of 7-(methacroyloxy)-2-oxo-heptylphosphonic acid and its copolymerization with methyl methacrylate

**DOI:** 10.1080/15685551.2019.1582216

**Published:** 2019-03-08

**Authors:** Štefan Chmela, Agnesa Fiedlerová, Tibor Liptaj, Yohann Catel, Norbert Moszner

**Affiliations:** a Polymer Institute, Slovak Academy of Sciences, Bratislava, Slovak Republic; b Central laboratories, Faculty of Chemical and Food Technology, Slovak University of Technology, Bratislava, Slovak Republic; c Ivoclar Vivadent AG, Schaan, Liechtenstein

**Keywords:** Adhesives, dental polymers, radical polymerizations, copolymerization parameters

## Abstract

The synthesis of polymerizable 7-(methacroyloxy)-2-oxo-heptylphosphonic acid M1 destined for self-etch adhesives is described. M1 is characterized by ^1^H, ^13^C and ^31^P-NMR spectroscopy. Its homopolymerization and copolymerization reactivity in the solvents methanol and dioxane between 45 and 70°C in the presence of azobisisobutyronitrile (AIBN) are examined. Polymerization proceeds readily through a thermal free radical initiation. The intensity exponents for the monomer and initiator are only slightly over 1 and approximately 0.5, respectively. This is in accordance with the results typically observed for an ideal free radical polymerization with termination mainly by disproportionation, which is typical for methyl methacrylate (MMA) homopolymerization. The kinetics of copolymerization with MMA are monitored by online ^1^H-NMR spectroscopy. Two copolymerization reactions for each pair of co-monomers are sufficient to evaluate the copolymerization parameters using the Jaacks method, the Fineman–Ross method and the nonlinear least-squares method. All three methods give similar results for particular monomer M1/MMA couple.

## 1. Introduction

**Scheme 1.**
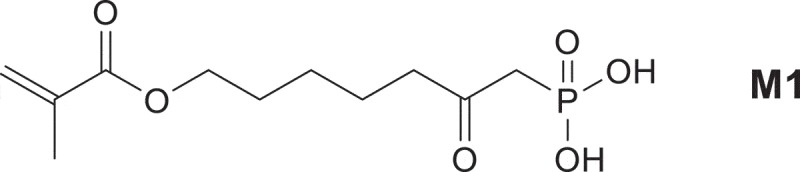
Structure of monomer **M1.**

Composite restorative materials represent success of modern biomaterials research since they replace biological tissue in both appearance and function [1]. Composite materials for dental restorations represent a unique class of biomaterials with severe restrictions on biocompatibility, curing behavior, esthetics, and ultimate material properties [1–3]. Generally, the composites are composed of three distinct phases, each with its own role in dictating material properties: the polymerizable resin, filler and the filler resin interface. Nowadays, self-etching adhesive developed for bonding of resin composite to enamel and dentin, containing phosphonic acid or dihydrogenphosphate groups have been quite widely considered [4–8]. Indeed, such derivatives are potentially interesting as the incorporation of a phosphonic or phophoric acid function would result in an increase of the biocompatibility and in the adhesion due to ionic interaction with calcium ions at the tooth surface [4] because of complex formation with calcium in hydroxyapatite [5]. This complex formation is acting as a macromolecular crosslinker [6] and the bond strength depends, for example, on the alkylen spacer length of the monomer [7,8]. In the most cases, they are monomers (methacrylates, methacrylamides, etc.) bearing phosphoric acid or phosphonic acid groups. High rate of homo and co-polymerization of all monomers is also a desired characteristic for dental materials. Therefore, it is important to understand the effect of molecular structure on polymerization reactivity. In this contribution, the synthesis of 7-(methacroyloxy)-2-oxo-heptylphosphonic acid **M1** is described and the free-radical homopolymerization in the protic solvent methanol and in dioxane solution is studied. The structure of given monomer **M1** is shown on Scheme 1.

The study of the copolymerization behavior of the monomer **M1** with methyl methacrylate (MMA) followed by ^1^H-NMR spectroscopy is also reported. The calculation of the copolymerization parameters was performed using the Jaacks method [9], the Fineman–Ross method [10] and the nonlinear least-squares method [11]. Copolymerization parameters for similar monomers as studied in this work were determined in our previous articles [12,13]. The reactivity ratios for the couple 10-(N-methylacrylamido)decylphosphonic acid (MADPA) and MMA were *r*_MADPA_ = 0.48 and *r*_MMA_ = 2.32. The reactivity ratios for the couple of diethyl 10-(N-methylacrylamido)-decylphosphonate (DEMADP) and MMA were *r*_DEMADP_ = 0.42 and *r*_MMA_ = 2.05. Methylacrylamides with long alkyl chains terminated with phosphonic acid or diethyl phosphonate groups are much less reactive than MMA. The nature of the functional groups (diethyl phosphonate or phosphonic acid) had no effect on the copolymerization kinetics. Different situation was found in the case of copolymerization of two methacrylates. The reactivity ratios for the couple 10-(methacryloyloxy)-decylphosphonic acid (MDPA) and MMA were *r*_MDPA_ = 1.01 and *r*_MMA_ = 0.95. The reactivity ratios for the couple of diethyl 9-(methacryloyloxy)-2-oxo-nonylphosphonate (DMONP) were *r*_DMONP_ = 1.28 and *r*_MMA_ = 1.08, for 9-(methacryloyloxy)-2-oxo-nonylphosphonic acid (MONA) *r*_MONA_ = 1.12 and *r*_MMA_ = 1.02 and for diethyl 9-(methacryloyloxy)-nonylphosphonate (DMONP) *r*_DMONP_ = 1.10 and *r*_MMA_ = 1.25.

Copolymerization of all four methacrylates bearing long alkyl chains with or without of 2-oxo group ended by phosphonate groups as well as free phosphonic acid with MMA provided very similar copolymerization parameters for all four pairs. All copolymerization parameters are close to 1 that proves the same reactivity of all four comonomers and MMA. This is in accordance with published data for different methacrylates [14]. MMA copolymerization reactivity ratios of MMA are similar as the reactivity ratios for ethyl methacrylate (EMA, *r*_MMA_ 1.08 – r_EMA_1.08; *r*_MMA_ 0.811 – r_EMA_ 0.86), propyl methacrylate (PMA, *r*_MMA_ 1.21 – r_PMA_ 1.24); n-butyl methacrylate (*r*_MMA_ 1.27 – r_BMA_ 1.2) and *iso*butyl methacrylate (iBMA, *r*_MMA_ 0.89 – r*_iBMA_* 1.2).

## 2. Experimental section

### 2.1. Abbreviations

Ammonium chloride (NH_4_Cl), azobisisobutyronitrile (AIBN), butylated hydroxytoluene (BHT), butyl lithium (BuLi), camphorquinone (CQ), dichloromethane (DCM), 4-dimethylaminopyridine (DMAP), methanol (MeOH), sodium sulfate (Na_2_SO_4_), tetrahydrofuran (THF), tetramethylsilane (TMS), trimethylsilyl bromide (TMSBr).

### 2.2. Materials

All reactions were carried out under an argon atmosphere. DCM and MeOH were dried over molecular sieves. Diethyl methylphosphonate was purchased from ABCR (Germany). All other reagents used in the syntheses were purchased from Sigma-Aldrich (Switzerland). TMSBr and methacrylic anhydride were distilled prior to use. Column chromatographies were performed on Macherey-Nagel silica gel 60 (40–63 µm).

### 2.3. Measurements

^1^H-NMR, ^13^C-NMR and ^31^P-NMR spectra were recorded on a DPX-400 spectrometer using TMS as internal reference for ^1^H-NMR and ^13^C-NMR chemical shifts and using H_3_PO_4_ (85%) as external reference for ^31^P-NMR chemical shifts. Data are given in the following order: chemical shift in ppm, multiplicity (s, singlet; bs, broad singlet; d, doublet; t, triplet; m, multiplet), coupling constant in Hertz, assignment.

#### 2.3.1. Diethyl 2-oxo-7-hydroxyheptylphosphonate (1)

n-BuLi (13.1 mL of a 2.5 M solution in hexane, 32.9 mmol) was added dropwise, at −78°C, to a solution of diethyl methylphosphonate (5.0 g, 32.9 mmol) in dry THF (80 mL). The reaction mixture was stirred for 1 h at −78°C. A solution of ε-caprolactone (2.5 g, 21.9 mmol) diluted in dry THF (40 mL) was added dropwise to the reaction mixture. The solution was stirred for 2 h at −78°C. Saturated NH_4_Cl aqueous solution (100 mL) and deionized water (50 mL) were slowly added to the reaction mixture. The solution was warmed up to room temperature and THF was removed under reduced pressure. The aqueous solution was extracted with DCM (3 × 200 mL). The combined organic layers were dried over Na_2_SO_4_ and concentrated under reduced pressure. The excess of diethyl methylphosphonate was removed under high vacuum (0.04 mbar). 5.83 g (21.9 mmol) of the desired phosphonate were isolated.

Yield: 100%. Aspect: colorless liquid. ^1^H-NMR (400 MHz, CDCl_3_, δ, ppm): 1.33 (t, ^3^J_HH_ = 7.0 Hz, 6H, POCH_2_CH_3_); 1.32–1.44 (m, 2H, CH_2_); 1.52–1.67 (m, 4H, CH_2_); 2.64 (t, ^3^J_HH_ = 7.2 Hz, 2H, CH_2_CH_2_CO); 3.07 (d, ^2^J_HP_ = 22.8 Hz, 2H, CH_2_P); 3.63 (t, ^3^J_HH_ = 6.5 Hz, 2H, CH_2_OH); 4.08–4.19 (m, 4H, POCH_2_CH_3_). ^31^P-NMR (162 MHz, CDCl_3_, δ, ppm): 19.9. ^13^C-NMR (101 MHz, CDCl_3_, δ, ppm): 16.3 (d, ^3^J_CP_ = 6.3 Hz, POCH_2_CH_3_); 23.0 (CH_2_); 25.0 (CH_2_); 32.3 (CH_2_); 42.3 (d, ^1^J_CP_ = 127.4 Hz, CH_2_P); 43.9 (CH_2_CH_2_CO); 62.3 (CH_2_OH); 62.7 (d, ^2^J_CP_ = 6.4 Hz, POCH_2_CH_3_); 202.1 (d, ^2^J_CP_ = 6.3 Hz, CO).

#### 2.3.2. Diethyl 2-oxo-7-(methacryloyloxy)heptylphosphonate (2)

Methacrylic anhydride (3.58 mL, 24.0 mmol) was added dropwise, under stirring, to a solution of the hydroxyphosphonate **1** (5.81 g, 21.8 mmol), triethylamine (3.35 mL, 24.0 mmol) and DMAP (133 mg, 1.1 mmol) in anhydrous DCM (60 mL). After stirring for 15 h, the solution was washed with deionized water (2 × 50 mL). The organic layer was dried over Na_2_SO_4_ and concentrated under reduced pressure. The crude product was purified by flash column chromatography (Eluent = hexane/ethyl acetate: 2/8). 5.88 g (17.6 mmol) of the desired phosphonate **2** were isolated.

Yield: 81%. Aspect: slightly yellow oil. ^1^H-NMR (400 MHz, CDCl_3_, δ, ppm): 1.33 (t, ^3^J_HH_ = 7.1 Hz, 6H, POCH_2_CH_3_); 1.32–1.43 (m, 2H, CH_2_); 1.57–1.73 (m, 4H, CH_2_); 1.91–1.95 (m, 3H, CH_3_); 2.64 (t, ^3^J_HH_ = 7.2 Hz, 2H, CH_2_CH_2_CO); 3.06 (d, ^2^J_HP_ = 22.9 Hz, 2H, CH_2_P); 4.08–4.19 (m, 6H, POCH_2_CH_3_ and CH_2_OCO); 5.52–5.56 (m, 1H, CH_2_ = C); 6.08 (bs, 1H, CH_2_ = C). ^31^P-NMR (162 MHz, CDCl_3_, δ, ppm): 19.9. ^13^C-NMR (101 MHz, CDCl_3_, δ, ppm): 16.3 (d, ^3^J_CP_ = 6.3 Hz, POCH_2_CH_3_); 18.3 (CH_3_); 23.0 (CH_2_); 25.4 (CH_2_); 28.4 (CH_2_); 42.4 (d, ^1^J_CP_ = 127.0 Hz, CH_2_P); 43.8 (CH_2_CH_2_CO); 62.5 (d, ^2^J_CP_ = 6.4 Hz, POCH_2_CH_3_); 64.4 (CH_2_OCO); 125.3 (CH_2_ = C); 136.4 (CH_2_ = C); 167.5 (CH_2_OCO); 201.8 (d, ^2^J_CP_ = 6.1 Hz, PCH_2_CO).

#### 2.3.3. 2-Oxo-7-(methacryloyloxy)heptylphosphonic acid (M1)

TMSBr (5.62 mL, 42.6 mmol) was added to a solution of phosphonate **2** (4.74 g, 14.2 mmol) in anhydrous DCM (50 mL). After stirring for 5 h at 30°C, the mixture was concentrated under reduced pressure. MeOH (50 mL) was added and the mixture was stirred for 30 min at RT. BHT (250 ppm) was added to the solution. The solvent was evaporated and the product was dried to a constant weight under vacuum. 3.86 g (13.9 mmol) of the desired phosphonic acid were isolated.

Yield: 98%. Aspect: light yellow solid. ^1^H-NMR (400 MHz, CDCl_3_, δ, ppm): 1.31–1.43 (m, 2H, CH_2_); 1.54–1.72 (m, 4H, CH_2_); 1.90–1.94 (m, 3H, CH_3_); 2.65 (t, ^3^J_HH_ = 7.2 Hz, 2H, CH_2_CH_2_CO); 3.20 (d, ^2^J_HP_ = 22.7 Hz, 2H, CH_2_P); 4.12 (t, ^3^J_HH_ = 6.8 Hz, 2H, CH_2_OCO); 5.53–5.57 (m, 1H, CH_2_ = C); 6.08 (bs, 1H, CH_2_ = C); 9.51 (bs, 1H, POH). ^31^P-NMR (162 MHz, CDCl_3_, δ, ppm): 21.6. ^13^C-NMR (101 MHz, CDCl_3_, δ, ppm): 18.3 (CH_3_); 22.8 (CH_2_); 25.2 (CH_2_); 28.3 (CH_2_); 42.5 (d, ^1^J_CP_ = 130.2 Hz, CH_2_P); 44.0 (CH_2_CH_2_CO); 64.7 (CH_2_OCO); 125.7 (CH_2_ = C); 136.3 (CH_2_ = C); 167.8 (CH_2_OCO); 204.5 (d, ^2^J_CP_ = 6.4 Hz, PCH_2_CO).

MMA was purified by distillation under reduced pressure and AIBN was recrystallized from methanol.

### 2.4. Monomer polymerization procedure

The basic parameters that control the radical polymerization of monomers **M1** was determined following their polymerization course at different monomer **M1** and initiator concentrations, and various temperatures. All polymerizations were performed in MeOH and dioxane. Monomer M1 is soluble in these solvents and there was not any precipitation even at very high conversions. The polymerization proceeds in a homogeneous medium.

Solution polymerizations proceeded in a series of 5–7 small glass ampoules. Approximately 0.50 mL of the polymerization batches filled each ampoule, which were later purged for 3 min with nitrogen before being sealed. The ampoules were removed one at a time from a thermostatic water bath at the desired time. The reaction was stopped by cooling the ampoules in ice water, and the addition of hydroquinone traces prevented post-polymerization.

### 2.5. Determination of monomer conversion

The monomer conversion in the case of homopolymerization was followed by FTIR spectroscopic determination of double bond consumption straight in monomer–polymer reaction mixtures in methanol. A NICOLET 8700 and NICOLET Impact 400 spectrophotometers from Thermo Fisher Scientific was used. Besides this the ATR FTIR with a Smart Orbit^TM^ single bounce ATR accessory and a Ge-crystal plate (resolution 4 cm^−1^, 64 scans) was used for double bond determination in residuum isolated from the reaction mixtures after MeOH or dioxane evaporation.

### 2.6. Copolymerization experiments

Monomers were dissolved in deuterated methanol and AIBN was used as a free radical initiator. After bubbling with nitrogen, the NMR tube was heated to 55°C and inserted into the preheated spectrometer (the temperature in the spectrometer was adjusted according to the ethylene glycol standard). ^1^H-NMR spectra were obtained on a Varian VNMRS 600 MHz spectrometer using the standard pulse programs and standard parameter settings provided by the manufacturer. The spectra were recorded as a function of time in 3-min intervals.

## 3. Results and discussion

### 3.1. Synthesis of monomer M1

**Scheme 2.**
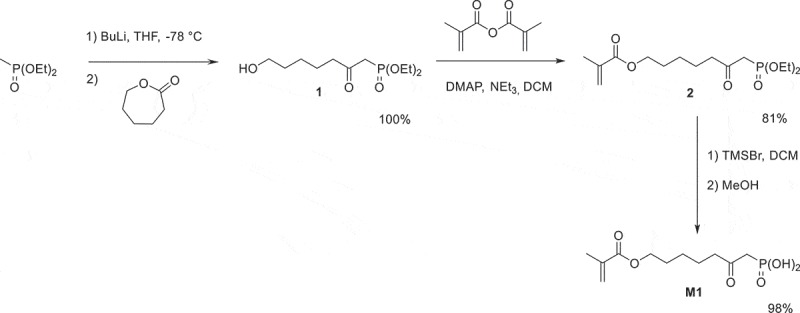
Synthesis of acidic monomer **M1.**

The acidic monomer **M1** was prepared in three steps, starting from diethyl methylphosphonate (Scheme 2). The ring opening of ε-caprolactone by diethyl lithiomethylphosphonate, followed by protonation, successfully afforded alcohol **1** in quantitative yield. Methacrylate **2** was subsequently synthesized by acylation of **1** with methacrylic anhydride in the presence of triethylamine and of a catalytic amount of DMAP. This monomer was obtained in 81% yield. The silylation of **2** using TMSBr, followed by the methanolysis of the silyl ether, finally provided the desired acidic monomer **M1** in 98% yield.

### 3.2. Free radical homopolymerization behavior of monomer M1

The kinetic features of interest concerning the free radical polymerization of monomer **M1** presented in this study are based on the analysis of the reaction course in the initial polymerization stages in MeOH or dioxane solution. The kinetic analysis is based on simplified assumptions that chain termination occurs only by a reaction between two propagating polymer radicals, and the system maintains steady-state reaction conditions. The instantaneous initial polymerizations rates Rp for the experiments with different monomer and initiator concentrations and at different temperatures were determined as a tangent in the origin of the conversion curves. They were used to evaluate the reaction intensity exponents for monomer and initiator and the overall Arrhenius activation energy. The survey of experimentally determined values is summarized in Table 1.

**Table 1.** Summary of kinetic characteristics for homopolymarization of monomer **M1**. Polymerizations in MeOH and dioxane initiated by AIBN.

Solvent		Methanol		Dioxane		
Batch Components in [mol/L]		Rp x 10^5^ [mol/Ls]	Intensity exponent	Rp x 10^5^ [mol/Ls]		Intensity exponent
[Monomer]: ([AIBN] = 0.05 mol/L, 60°C)	0.25	4.1	to **M1** 1.35	4.5		to **M1** 1.05
	0.50	10.6		10.3		
	0.75	17.8		14.6		
[AIBN]: ([Monomer] = 0.5 mol/L, 60°C)	0.025	7.5	to **AIBN** 0.44	7.5		to **AIBN** 0.49
	0.05	10.6		10.3		
	0.075	12.0		13.1		
Temperature [°C]: ([Monomer] = 0.5 mol/L, [AIBN] = 0.05 mol/L)	45	2.88	Ea = 73.2 [kJ/mol]	50	3.1	Ea = 71.5 [kJ/mol]
	55	6.9		60	10.3	
	65	15.0		70	17.7	

#### 3.2.1. Conversion-time dependences for monomer M1 in methanol

**Figure 1.**
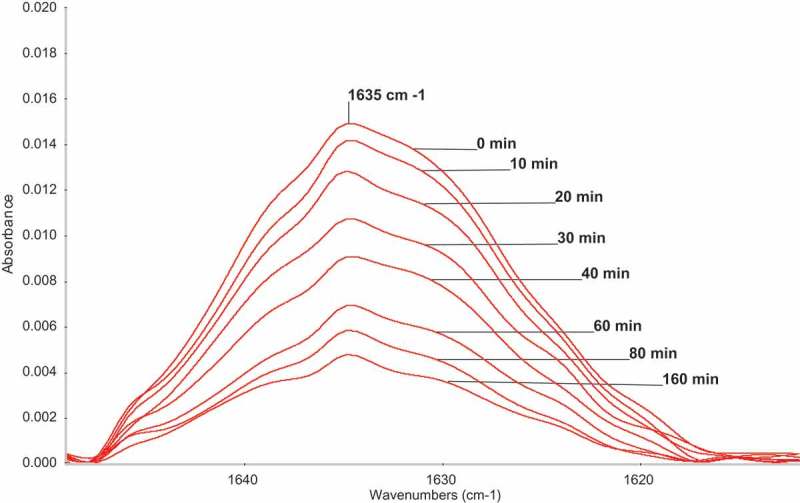
Changes of the part of ATR-FTIR spectra of **M1** during homopolymerization. 0.25 mol/L **M1**; 0.025 mol/L AIBN; MeOH; 60°C.

**M1** was polymerized in MeOH, at 60°C, in three different concentrations: 0.25, 0.5, and 1.0 mol/L. AIBN (0.025 mol/L) was used as an initiator. Conversion was followed by FTIR measurements. After polymerization, MeOH was evaporated and ATR FTIR spectra were measured. The changes of the part of FTIR spectra are shown in Figure 1.

Conversions were calculated from the height as well as from integral intensities of the relatively broad peaks at 1635 cm^−1^ belonging to stretching vibration of double bonds.

**Figure 2.**
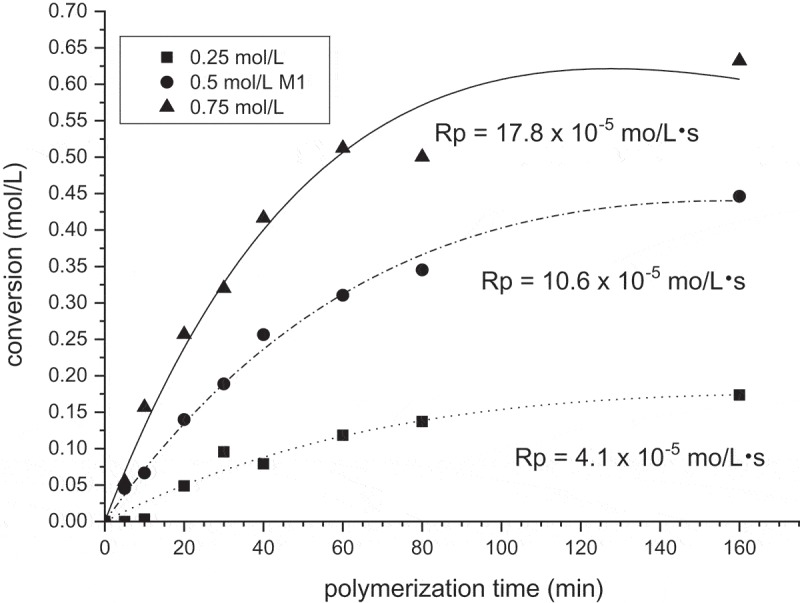
Monomer conversion of **M1** during homopolymerization. [AIBN] = 0,05 mol/L, *T* = 60°C, MeOH.

Effect of the monomer concentration on the course of polymerization is shown in Figure 2 in the form of conversion-time dependences for 0.25, 0.5, and 0.75 mol/L monomer concentrations. The polymerization is very fast from the beginning without any induction period. Reflection of this is high initial polymerization rates. The following values of Rp were obtained: for concentration of **M1** 0.25 mol/L Rp was 4.1 × 10^−5^ mol/L·s, for 0.5 mol/L Rp = 10.6 x10^−5^ mol/L·s and for 0.75 mol/L Rp = 17.8 x10^−5^ mol/L·s.

**Figure 3.**
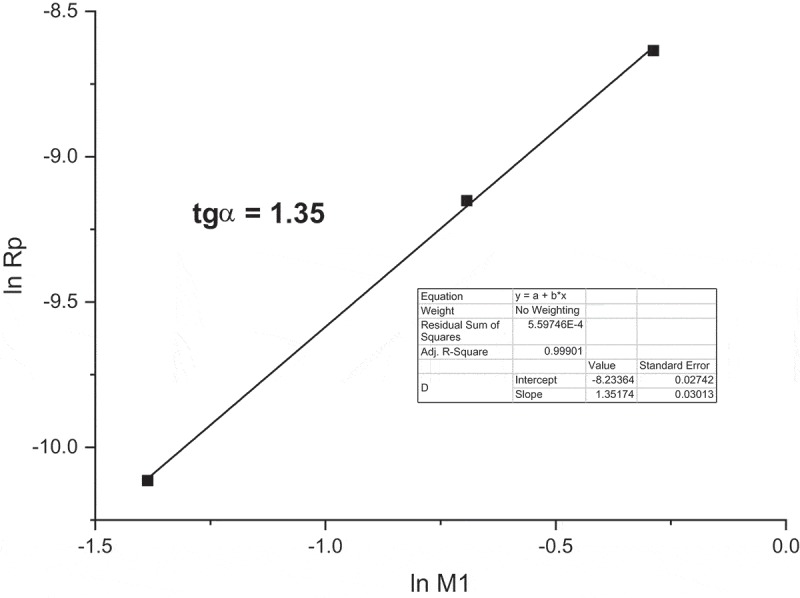
Dependence of **M1** initial polymerization rates on starting monomer concentrations in MeOH at 60°C; [AIBN] = 0.05 mol/L.

The kinetic theory predicts Rp = K [I]^0.5^. [M]^1.0^. By plotting ln Rp against ln [**M1**] concentration (Figure 3), the intensity exponent to monomer concentration was determined. The experimental points are satisfactory correlated with line and the slope 1.35 is higher than the theoretical value 1. Nevertheless, if we admit that the value 1.35 lays of experimental uncertainty we should suppose that termination depends on length of the growing polymer chains or does not proceed solely by the mutual interaction of polymer radicals. The initiator primary radicals probably contribute to radical termination.

**Figure 4.**
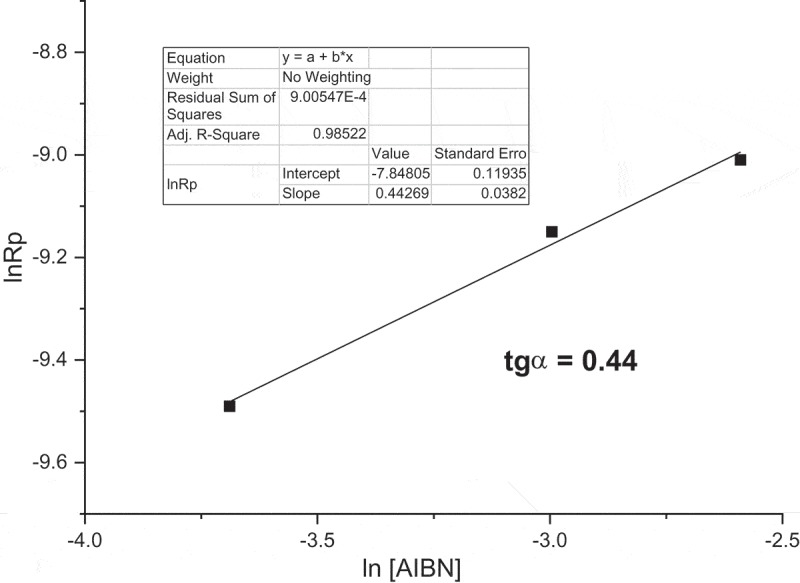
**M1** initial polymerization rates in dependence on AIBN initiator concentrations (60°C; monomer concentration 0.5 mol/L; MeOH).

The polymerization of **M1** was subsequently performed at 60°C using three different concentrations of AIBN. Similarly as in the previous case, the polymerization rates Rp were calculated. Polymerization rates Rp at 60 ⁰C in dependence on 0.025, 0.05, 0.075 [mol/L] of AIBN at 0.5 [mol/L] monomer concentrations shown in Figure S1 in Supplementary Material.

confirm standard polymerization behavior for **M1** monomer. The logarithmic dependence of the Rp on AIBN concentration, as shown in Figure 4, is linear with the slope 0.44.

The coefficient is a little lower than predicted value 0.5 but the deviation is small enough to accept typical behavior for free radical polymerization kinetic with prevailing termination mainly by disproportionation.

The last studied dependency was the effect of temperature on the course of **M1** homopolymerization. The courses of **M1** conversion during polymerization at 45, 55, and 65 ⁰C (0.05 mol/L AIBN and 0.5 mol/L of monomer) shown in Figure S2 in Supplementary Material indicate that conversion started from the beginning of polymerization for the all temperatures. The natural logarithm of the effective reaction rate Rp was plotted versus reciprocal absolute temperature (Figure S3 in Supplementary Material) indicating an Arrhenius temperature dependence. An apparent activation energy of 73.2 kJ/mol is obtained. This value is a little bit lower than the values for bulk polymerization of MMA. Literature reports values ranging from 78 to 86 kJ/mol [15].

#### 3.2.2. Conversion-time dependences for monomer M1 in dioxane

**Figure 5.**
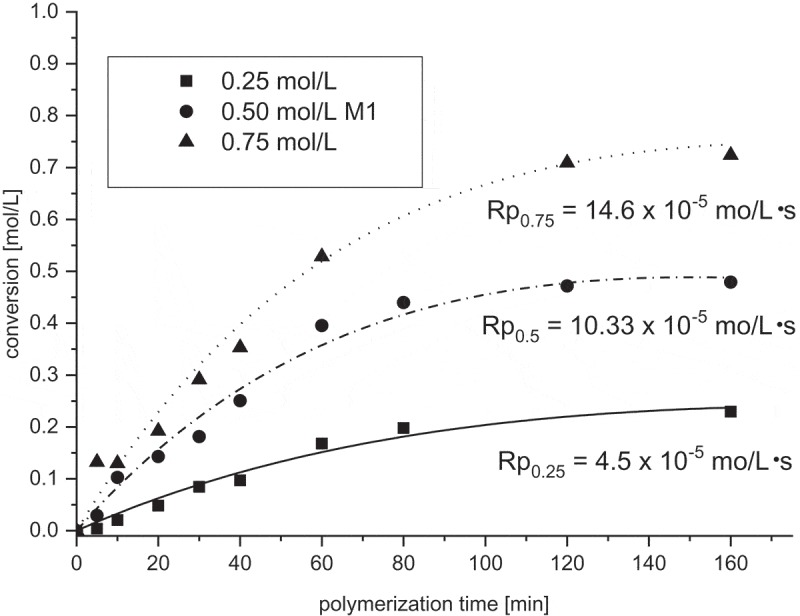
Monomer conversion of **M1** during homopolymerization in dioxane. [AIBN] = 0,05 mol/L, T = 60°C.

Similar experiments concerning the effect of monomer and AIBN concentration as well as effect of temperature on the rate of **M1** homopolymerization were performed in the non-protic solvent dioxane. Conversions as in the previous MeOH case were calculated from the height as well as from integrals of the relatively broad peaks at 1635 cm^−1^ belonging to symmetric vibration of double bond measured by ATR FTIR from the samples obtained from residues after dioxane evaporation. The effect of **M1** concentration on the **M1** conversion during polymerization in dioxane is shown in Figure 5.

**Figure 6.**
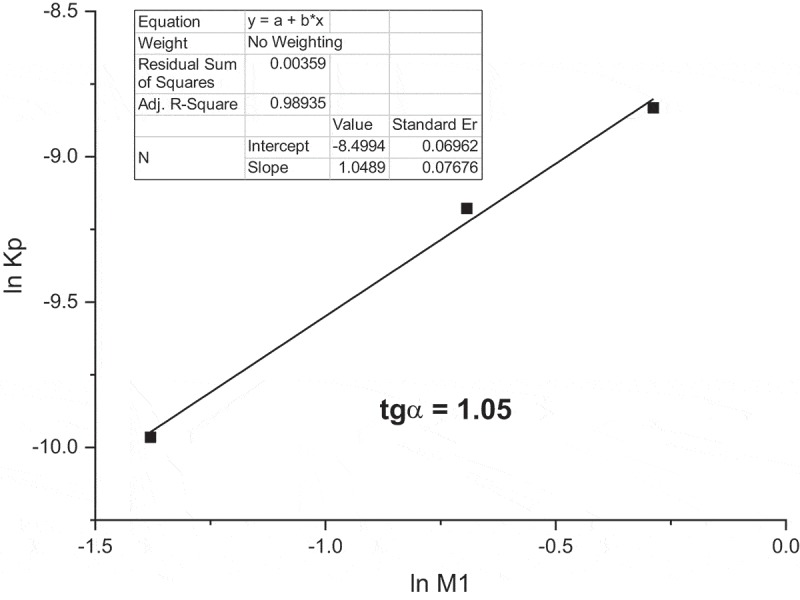
**M1** initial polymerization rates in dependence on monomer concentration in the Arrhenius coordinates ([AIBN] = 0.05 mol/L, 60°C, dioxane).

The calculated Rp are very similar as in the case of methanol shown in Figure 2. The dependency of ln [M1] on ln Rp satisfactory correlated well with straight line (Figure 6). The slope 1.05 corresponding to the intensity exponent to monomer concentration is very similar with predicted value 1 for typical free radical homopolymerization.

The polymerization of **M1** was subsequently performed in dioxane at 60°C using three different concentrations of AIBN. Conversions of **M1** at 60 ⁰C for three AIBN concentrations (0.025, 0.05, 0.075 mol/L) at 0.5 [mol/L] monomer concentrations shown in Figure S4 in Supplementary Material confirm standard polymerization behavior for **M1** monomer. The dependence of the ln Rp on ln [AIBN] concentration, shown in Figure S5 in Supplementary Material, is linear with the slope 0.49. As in the case of the intensity exponent for monomer

concentration, the intensity exponent for AIBN concentration – 0.49 is almost exactly the same as for predicted value 0.5 for ideal free radical polymerization.

The last studied dependency was the effect of temperature on the course of **M1** homopolymerization in dioxane. The courses of **M1** conversion during polymerization at 50, 60 and 70 ⁰C (0.05 mol/L AIBN and 0.5 mol/L of monomer) showed that conversion started from beginning of polymerization for the all three temperatures (Figure S6 in Supplementary Material). The natural logarithm of the effective reaction rate Rp was plotted versus reciprocal absolute temperature indicating an Arrhenius temperature dependence. Apparent activation energy of 71.5 kJ/mol is obtained (Figure S7 in Supplementary Material).

As shown in Table 1, we can conclude that in all cases we are dealing with typical standard processes abide by the generally accepted rules for typical free radical polymerization. The solvent effect on the kinetics of the radical polymerization was first time mentioned in pioneering work of Burnett et al. [16]. Since then many studies have shown that initiation rate is not affected by the presence of different solvents [17–19] and termination rate appears to be proportional to the medium viscosity [20,21]. Concerning the propagation rate constant, the small effect has been attributed to complexing of the propagation radical with solvent. All these data were based on rate measurements from rotating sector yielding k_p_/k_t_ combined with k^2^_p_/k_t_ data from stationary measurements. Utilization of a rather convenient PLP/SEC method (Pulsed Laser Polymerization combined with a subsequent analysis of the chain length distribution of the resulting polymer by Size Exclusion Chromatography) [22,23] permits direct measuring of k_p_. Using of this method it has been shown that k_p_ for MMA polymerization in different solvents is fairly the same as observed in bulk polymerization [24]. Kinetic characteristics shown in Table 1 are in line with this results and there is no remarkable changes between the polymerization in MeOH and dioxane.

## 4. Free radical copolymerization behavior of monomers M1 with MMA

### 4.1. Analysis of the copolymerization kinetics followed by ^1^H-NMR spectroscopy: evaluation of the copolymerization parameters

The copolymerizations of MMA with **M1** were followed by online ^1^H-NMR spectroscopy. In this method, the ^1^H-NMR spectroscopic data of the kinetics are evaluated in way that a set of two subsequent NMR spectroscopic measurements at different times are taken as a single kinetic experiment. The first NMR spectroscopic measurement at time *t* gives the feed composition ([M_1_], [M_2_]). The monomer consumption between the first and second measurements – *t *+ *x* (the conversion is roughly 5%) gives the comonomer ratio in the polymer (Δ [M_1_], Δ [M_2_]). The second NMR spectroscopic measurement at time *t + x* gives the final composition as well as the new feed composition at time *t + x*. The monomer reactivity ratios were obtained by Jaacks [9] method using higher monomer feed ratios (MMA:**M1** = 3 and 0.3, respectively) and the Fineman–Ross method [10]. The last method for evaluation of the copolymerization parameters is the nonlinear least-squares method [11]. For better accuracy of the results, copolymerization experiments were realized directly in the spectrometer.

#### 4.1.1. Copolymerization of MMA with M1

**Figure 7.**
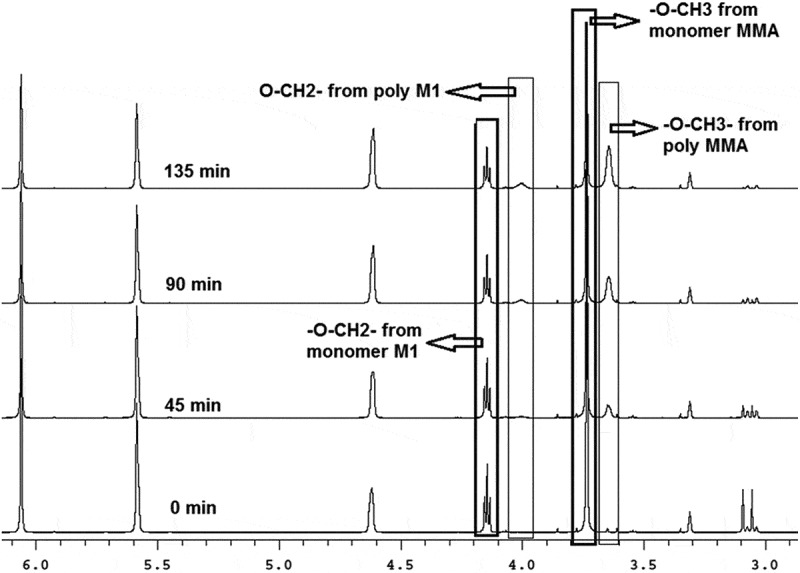
Details of the ^1^H-NMR spectra of the copolymerization of **M1** with MMA at various time in deuterated methanol, T = 55°C. Molar ratio **M1**:MMA = 3.65:1 total monomers concentration = 0.5 mol/L, [AIBN] = 0.025 mol/L, CDCl_3._

The changes of the ^1^H-NMR spectra of the mixture **M1**:MMA = 3.65:1 during copolymerization in deuterated chloroform measured directly in NMR spectrometer are shown in Figure 7. There are two non-equivalent olefinic hydrogens from MMA as well as from **M1** represented by two singlets and possessing exactly the same chemical shifts at 5.58 and 6.08 ppm for both co-monomers. Therefore, they are useless for differentiation between the rate of consummation of the two monomers during copolymerization. The total conversion can be calculated based on these peaks. The methyl ester group from MMA monomer appears as a sharp singlet at 3.72 ppm, while this group in copolymer appears at 3.63 ppm as a broader singlet. The triplet of O-CH_2_- group in monomer **M1** appears at 4.14 ppm while in polymer it changes to broad singlet at 3.95 ppm. These four sufficiently separated peaks allow us to follow kinetics of copolymerizations as well as composition of copolymers. The integral intensities of these peaks are proportional to the monomers concentration during the copolymerization and their time evolutions were evaluated as the function of the feed composition at the beginning as well as during the copolymerization.

**Figure 8.**
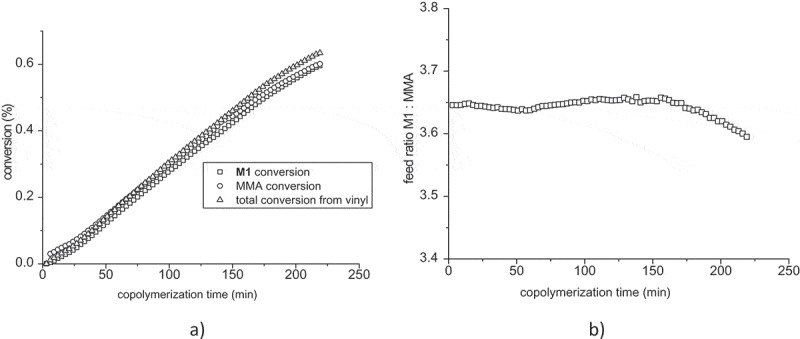
(a) Monomer conversions of **M1**, MMA and total conversion during the copolymerization of mixture **M1**: MMA = 3.65:1. (b) comonomer ratio **M1**: MMA in feed versus time.

The courses of **M1**, MMA and total conversions are shown in Figure 8(a). The conversion of MMA is almost the same as the conversion of **M1**. It is copolymerization of two mathacrylates with different ester group. **M1** represents methacrylate with 2-oxo-heptylphosphonic acid. Methyl group in MMA as well as long alkyl chain containing keto group and free phosphonic acid seems to have the same electronic effect on the vinyl groups of comonomers. The reactivity of both possible grooving macroradicals with both comonomers is the same. Consequently, the comonomer ratio of **M1**: MMA is almost constant during the copolymerization as it is shown in Figure 8(b) for starting ratio **M1**: MMA = 3.65.

Quite similar situation was in the case of MMA excess at molar ratio **M1**: MMA = 1:3.18 (Figure S8a in Supplementary Material). The difference between conversion of **M1** and MMA was much smaller. Similarly, the changes of feed ratio shown in Figure S8b in Supplementary Material are less marked as in the previous case.

**Figure 9.**
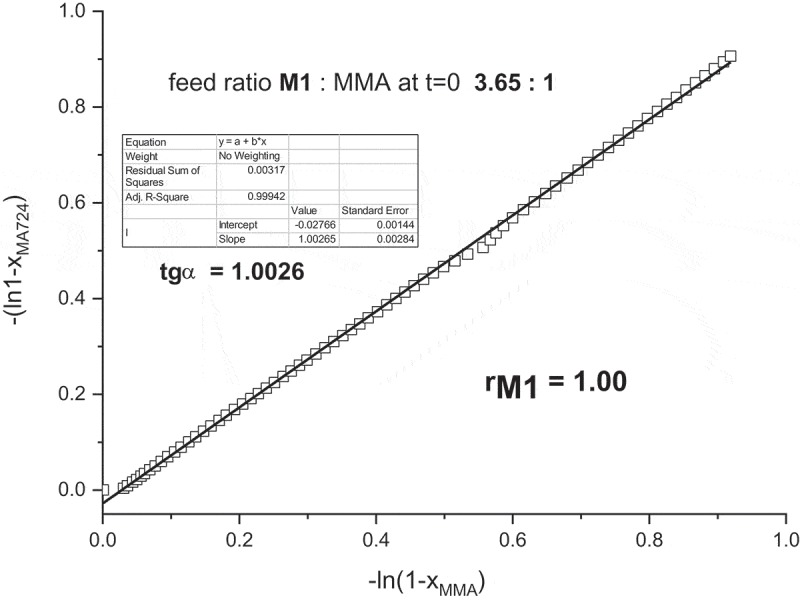
Jaacks plot obtained from time-conversion plot in Figure 8 (a).

The first used method for determination of reactivity ratio is Jaacks method [8]. The slope of dependency of the rate of **M1** consumption against the rate of MMA consumption gives the

reactivity ratio *r_M1_*, as shown in Figure 9. The reactivity ratio *r**_M1_*** is 1.00. The reactivity ratio *r*_MMA_ was obtained from copolymerization with excess of **M1** and Jacks plot is shown in Figure S9 in Supplementary Material. The received copolymerization parameter was *r*_MMA_ = 0.95.

**Figure 10.**
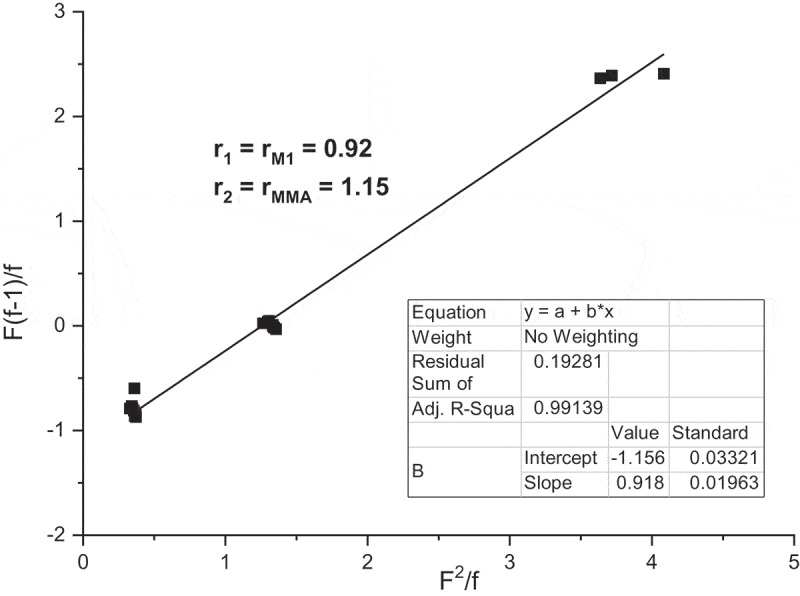
Fineman–Ross plot for the free radical copolymerization of **M1** and MMA.

The Fineman–Ross method has been applied for numerous systems. This method is based on linearization of the dependence of *F* = [*M*_1_]/[*M*_2_] versus f = [m_1_]/[m_2_]. [M_1_]/[M_2_] is the molar ratio of monomer units in feed and [m_1_]/[m_2_] is the molar ratio of monomer units in copolymer [10]. The Fineman–Ross plot graphs *F*(*f*–1)/*f* versus *F*^2^/f for the copolymerization of **M1** with MMA is shown in Figure 10. The received copolymerization parameters were *r***_M1_** = 0.92 and *r*_MMA_ = 1.15. The value is a little lower than the value obtained from Jaacks method for **M1** – r_M1_ = 1.00 and higher in MMA *r*_MMA_ = 1.15 as compared with *r*_MMA_ = 0.92 but the differences are very small especially for **M1**.

The last method for evaluation of the copolymerization parameters is the nonlinear least-squares method. The copolymerization diagram plots the instantaneous copolymer composition F as a function of the initial feed composition *f* (*t* = 0). The variables are defined as follows:

$$f_{1}=1-f_{2}=\frac{\left[M_{1}\right]}{\left[M_{1}\right]+\left[M_{2}\right]}$$

$$F_{1}=1-F_{2}=\frac{r_{1}f_{1}^{2}+f_{1}f_{2}}{r_{1}f_{1}^{2}+2f_{1}f+r_{2}f_{2}^{2}}$$

**Figure 11.**
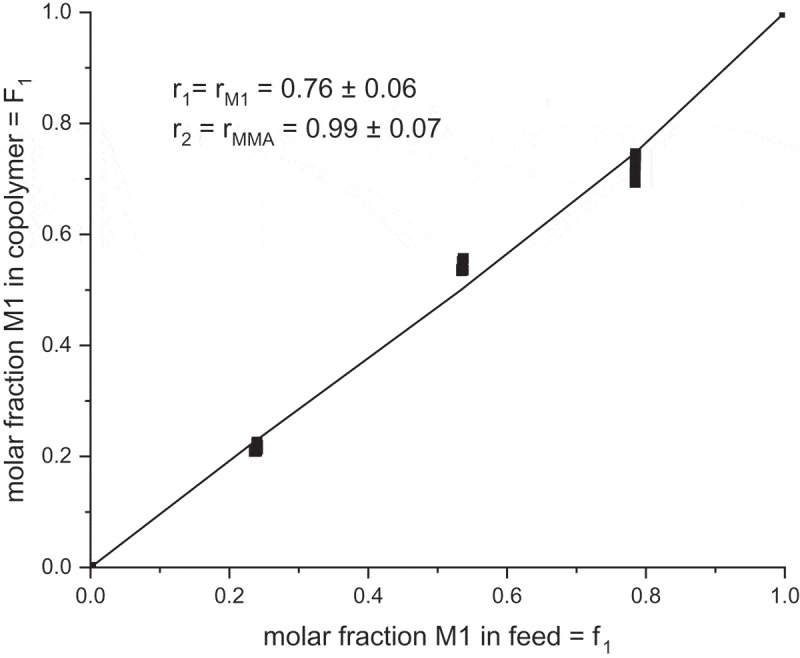
Instantaneous copolymer composition *F*_1_ versus commoner feed composition *f*_1_ of **M1** in copolymerization with MMA; fit of the data points to *r*_1_ = r**_M1_** and *r*_2_ = *r*_MMA_.

The data from the kinetic experiments can be fit with these equations with the nonlinear least-squares method [11]. The fit is shown in Figure 11 and the resulting *r* parameters are in very good agreement with those values from Fineman–Ross method: r**_M1_** = 0.76 and *r*_MMA_ = 0.99. Both parameters are lower than one. It means that grooving macro radical with the ultimate **M1** structure prefers reaction with MMA. A little bit heterogeneous sample is expected from the free radical copolymerization of **M1** with MMA. The reactivity ratios of **M1** and MMA determined by different methods are summarized in Table 2.

**Table 2.** Reactivity ratio of **M1** and MMA determined by different methods.

Method	*r*_M1_	*r*_MMA_	*r*_M1_ . *r*_MMA_
Fineman–Ross	0.92	1.15	1.06
Nonlinear least squares	0.76	0.99	0.75
Jaacks	1.00	0.95	0.95
Average value	0.89	1.03	0.92

## 5. Conclusions

The basic kinetic characteristics of the free radical homopolymerization of **M1** shows that we are dealing with typical standard processes abide by the generally accepted rules for typical free radical polymerization. There are no remarkable changes between the polymerization in MeOH and dioxane.

Concerning the copolymerization of **M1** with MMA, we can conclude that copolymerization provides very similar copolymerization parameters for all three used methods. From the courses of copolymerization for **M1** and MMA, it follows that there is a little bit faster consumption of MMA than consumption of **M1**. The Fineman–Ross and nonlinear least-squares method revealed a little bit higher copolymerization parameter for MMA (1.15; 0.99) than for **M1** (0.92; 0.76). Jaacks method provides almost the same values for **M1** (1.00) as well as for MMA (0.95). It is possible to summarize that we are dealing with statistical copolymerization and the composition of copolymers is practically the same as the composition of feeds for all conversions.

## References

[1] Cramer NB, Stansbury JW, Bowman CN. Recent advances and developments in composite dental restorative materials. J Dent Res. 2011;90:402–416. 20924063 10.1177/0022034510381263 PMC3144137

[2] Moszner N, Hirt T New polymer‐chemical developments in clinical dental polymer materials: enamel–dentin adhesives and restorative composites. J Pol Sci, Part A Polym Chem. 2012;50:4369–4402.

[3] Shalaby WS, Salz U Polymers for dental and orthopaedic applications. Boca Raton: CRC Press, Taylor & Francis Group; 2007 p. 13–168.

[4] Mou LY, Singh G, Nicholson JW Synthesis of a hydrophilic phosphonic acid monomer for dental materials. Chem Commun. 2000;5:345–346.

[5] Avci D, Mathias LJ Synthesis and photopolymerizations of phosphate-containing acrylate/(di)methacrylate monomers from 3-(acryloyloxy)-2-hydroxypropyl methacrylate. Polym Bull. 2005;54(1–2):11–19.

[6] Bayle MA, Gregoire G, Sharrock P The role of acrylophosphonic acid monomers in the formation of hybrid layers based on self-etch adhesives. J Dent. 2007;35(4):302–308. 17113698 10.1016/j.jdent.2006.10.006

[7] Nishiyama N, Aida M, Fujita K, et al NMR study on the adhesion efficacy of experimental phosphonic acid monomers. Dent Mater J. 2007;26(3):382–387. 17694748 10.4012/dmj.26.382

[8] Van Landuyt KL, Yoshida Y, Hirata I, et al Influence of the chemical structure of functional monomers on their adhesive performance. J Dent Res. 2008;87(8):757–761. 18650548 10.1177/154405910808700804

[9] Jaacks V A novel method of determination of reactivity ratios in binary and ternary copolymerizations. Makromol Chem. 1972;161(1):161–172.

[10] Fineman M, Ross SD Linear method for determining monomer reactivity ratios in copolymerization. J Pol Sci. 1950;V(2):259–262.

[11] Tidwell PV, Mortimer GA An improved method of calculating copolymerization reactivity ratios. J Pol Sci Part A Gen Pap. 1965;3:369–387.

[12] Chmela Š, Pavlinec J, Fiedlerová A, et al Determination of homopolymerization kinetics of 10-(N-methylacrylamido)-decylphosphonic acid, its diethyl ester, and 10-(methacryloyloxy)-decylphosphonic acid, and their copolymerization with methyl methacrylate. Macromol Chem Phys. 2015;216(24):2386–2397.

[13] Chmela Š, Fiedlerová A, Liptaj T, et al Determination of homopolymerization kinetics and copolymerization with methyl methacrylate of diethyl 9-(methacryloyloxy)-2-oxo-nonylphosphonate, 9-(methacryloyloxy)-2-oxo-nonylphosphonic acid and diethyl 9-(methacryloyloxy)-nonylphosphonate. e-Polymers. 2018;18(3):205–216.

[14] Greenlay RZ Polymer Handbook In: Brandrup J, Immergut EH, Grulke EA, editors. 4th ed. New York (NY): Wiley; 1999 p. II/232.

[15] Greenlay RZ Polymer Handbook In: Brandrup J, Immergut EH, Grulke EA, editors. 4th ed. New York (NY): Wiley; 1999 p. II/415.

[16] Bumett GM, Dailey WS, Pearson JM Radical polymerization in halogenated solvents. part 1. methyl methacrylate polymerization initiated by azoisobutyronitrile. Trans Faraday Soc. 1965;61:1216–1225.

[17] Kamachi M Influence of solvent on free radical polymerization of vinyl compounds. Ad Polym Sci. 1981;38:55–87.

[18] Bamford CH, Brumby S The effect of aromatic solvents on the absolute rate coefficients in the polymerization of methyl methacrylate at 25°C. Makromol Chem. 1967;105:122–131.

[19] Kamachi M, Satoh J, Liaw DJ, et al Solvent effects on radical polymerization of vinyl benzoate and phenyl methacrylate. Macromolecules. 1977;10(2):501–502.

[20] Burnett GM, Cameron GG, Joiner SN Solvent effects on the free radical polymerization of styrene. J Chem Soc Faraday Trans 1. 1973;69:322–327.

[21] Kamachi M, Liaw DJ, Nozakura S Solvent effect on radical polymerization of phenyl methacrylate. Polym J. 1977;9(3):307–316.

[22] Olaj OF, Bitai I, Hinkelmann F The laser-flash-initiated polymerization as a tool of evaluating kinetic constants of free radical polymerization. 2. The direct determination of the rate constant of chain propagation. Makromol Chem. 1987;188:1689–1702.

[23] Olaj OF, Bitai I The laser flash‐initiated polymerization as a tool of evaluating (individual) kinetic constants of free radical polymerization, 3. Information from degrees of polymerization. Angew Makromol Chem. 1987;155(1):177–190.

[24] Olaj OF, Bitai I Solvent effects on the rate constant of chain propagation in free radical polymerization. Monats Chem. 1999;130(6):731–740.

