# Supplementary material for: Synthesis and homopolymerization kinetics of 7-(methacroyloxy)-2-oxo-heptylphosphonic acid and its copolymerization with methyl methacrylate
Source: Des Monomers Polym. 2019 Mar 8;22(1):79–90. doi: 10.1080/15685551.2019.1582216 (PMC6419686; doi:10.1080/15685551.2019.1582216)
Supplement: Supplemental Material [file TDMP_A_1582216_SM1781.docx]

**Supplementary Information**

Figure S1. Conversion of monomer **M1** at different AIBN concentrations. T = 60 ^o^C; [**M1**] = 0.5 mol/L, methanol.

Figure S2. Conversion of **M1** – in the dependence of the polymerization temperature. [**M1**] = 0.5 mol/L; [AIBN] = 0.05 mol/L, MeOH.


Figure S3. **M1** initial polymerization rates in dependence on temperature in the Arrhenius plot ([AIBN] = 0.05 mol/L; [**M1**] = 0.5 mol/L; MeOH).

Figure S4. Conversion of monomer **M1** at different AIBN concentrations. T = 60 ^o^C; [**M1**] = 0.5 mol/L, dioxane.


Figure S5. **M1** initial polymerization rates in dependence on AIBN concentration in the Arrhenius coordinates ([**M1**] = 0.5 mol/L, 60 ^o^C, dioxane).

Figure S6. Conversion of **M1** –in the dependence of the polymerization temperature. [**M1**] = 0.5 mol/L; [AIBN] = 0.05 mol/L, dioxane.

Figure S7. **M1** initial polymerization rates in dependence on temperature in the Arrhenius plot ([AIBN] = 0.05 mol/L; [M1] = 0.5 mol/L; dioxane).

a) b)

Figure S8. a) Monomer conversions of **M1**, MMA and total conversion during the copolymerization of mixture **M1** : MMA = 1 : 3.18; (b) comonomer ratio **M1** : MMA in feed versus time.

Figure S9 . Jaacks plot obtained from time-conversion plot in Figure S8a).
